# SARS-CoV-2 Gastrointestinal Infection Prolongs the Time to Recover From COVID-19

**DOI:** 10.3389/fmed.2021.683551

**Published:** 2021-06-04

**Authors:** Zhijie Xu, Meiwen Tang, Ping Chen, Hongyu Cai, Fei Xiao

**Affiliations:** ^1^Department of Infectious Diseases, The Fifth Affiliated Hospital of Sun Yat-Sen University, Zhuhai, China; ^2^Guangdong Provincial key Laboratory of Biomedical Imaging, The Fifth Affiliated Hospital of Sun Yat-Sen University, Zhuhai, China; ^3^Guangdong Provincial Engineering Research Center of Molecular Imaging, The Fifth Affiliated Hospital of Sun Yat-Sen University, Zhuhai, China; ^4^Department of Hematology, The Fifth Affiliated Hospital of Sun Yat-Sen University, Zhuhai, China

**Keywords:** SARS-CoV-2, gastrointestinal infection, adverse effect, stool viral load, alimentary tract

## Abstract

**Objectives:** We previously reported that SARS-CoV-2 infects the gastrointestinal (GI) epithelium. In this study, we aimed to explore the impact of SARS-CoV-2 GI infection on clinical outcomes of COVID-19.

**Materials and Methods:** For this retrospective cohort study, 104 patients with COVID-19 were classified into a SARS-CoV-2 GI infection group and a non-infection group. The primary endpoint was the time of negative conversion of SARS-CoV-2 RNA in respiratory tract samples. The secondary outcome was the time of hospitalization for COVID-19.

**Results:** Patients with SARS-CoV-2 GI infection had a longer duration of positive SARS-CoV-2 RNA in respiratory tract samples (median 12.0 days [95% CI: 10.0–13.2] vs. 9.0 days [95% CI: 7.5–10.5]; HR 0.575 [95% CI: 0.386–0.857]; *P* = 0.003) and hospitalization (median 28.0 days [95% CI: 23.2–32.8] vs. 15.0 days [95% CI: 13.6–16.4]; HR 0.149 [95% CI: 0.087–0.252]; *P* < 0.001) than patients without SARS-CoV-2 GI infection. Subgroup analyses for sex, age, epidemiological history, clinical classification and antiviral treatment showed consistent results.

**Conclusion:** Our study indicates that SARS-CoV-2 GI infection prolongs the duration of SARS-CoV-2 shedding and hospitalization in the patients with COVID-19. More attention should be paid to SARS-CoV-2 GI infection of COVID-19 and fecal SARS-CoV-2 RNA test should be completed in time.

## Introduction

COVID-19, which is caused by SARS-CoV-2, is a global pandemic resulting in millions of deaths (1). The virus is mainly transmitted via respiratory droplets and usually causes a variety of respiratory symptoms. Various lines of evidence indicate that SARS-CoV-2 also infects the gastrointestinal (GI) system and causes corresponding GI symptoms (2). We previously reported that SARS-CoV-2 infects the GI epithelium by detecting positive staining for SARS-CoV-2 in the GI epithelium of patients positive for fecal SARS-CoV-2 RNA (3). Several laboratories around the world have also detected the virus in fecal samples, in GI biopsies and at autopsy (4–6). Subsequently, more attention is being given to the GI manifestations of SARS-CoV-2. The most common GI symptoms of COVID-19 are anorexia, diarrhea, nausea and abdominal pain (5, 7, 8).

Several studies have investigated the relationship between GI symptoms and mortality of COVID-19 (9–11), however, no studies have described associations between SARS-CoV-2 GI infection and the clinical outcomes of COVID-19. Thus, to further explore the clinical characteristics of SARS-CoV-2 GI infection in patients with COVID-19, we aimed to explore its impact on clinical COVID-19 outcomes in a retrospective cohort study.

## Methods

### Patients

This retrospective cohort study was conducted from 18 January to 24 August 2020 at the Fifth Affiliated Hospital of Sun Yat-Sen University (SYSU5) in Zhuhai, China. We recruited 105 patients who were hospitalized in the COVID-19 medical center of SYSU5. The diagnosis of COVID-19 was based on epidemiological history, symptoms, lung imaging manifestations and the presence of SARS-CoV-2 RNA in respiratory tract samples using the China CDC-standardized real-time reverse transcriptase polymerase chain reaction (rRT-PCR). The copy numbers of SARS-CoV-2 RNA were indicated by rRT-PCR cycle threshold (CT) values, with lower CT values corresponding to higher viral copy numbers. A CT value <40 was defined as positive, as reported (12). GI infection of SARS-CoV-2 was defined as simultaneously positivity for SARS-CoV-2 RNA in a fecal sample. After excluding one patient for whom the fecal SARS-CoV-2 RNA test was not completed, 104 patients were included and divided into two groups according to whether SARS-CoV-2 GI infection was present (infection group; *n* = 54) or absent (non-infection group; *n* = 50).

This study was approved by the Ethics Committee of SYSU5 [No. ZDWY [2020] Lunzi No. (K22-1)]. All experiments in this study were performed according to the principles of the Declaration of Helsinki and Good Clinical Practice. All patients or their legal guardians provided written informed consent.

### Data Collection and Measurement

Information about sex, age, ethnicity, smoking, alcohol consumption, epidemiological history, medical history, clinical classification, and antiviral treatment was obtained from the hospital information system of SYSU5. Epidemiological history was recorded as local or overseas cases according to the possible location of infection with SARS-CoV-2. In the present study, local cases had travel history to Wuhan or contact with patients with COVID-19 or other person with fever or respiratory symptoms from Wuhan, and no overseas travel history within 14 days before illness onset. Overseas cases had overseas travel or residence history and tested positive for SARS-CoV-2 RNA in respiratory tract samples when they entered China. The identification of hypertension and diabetes was based on previous relevant medical history or diagnoses during hospitalization. Clinical classification was classified as mild (mild clinical symptoms and absent of pneumonia manifestations on imaging), moderate (obvious symptoms and present of pneumonia manifestations on imaging), severe (respiratory rate ≥30 breaths/min; oxygen saturation ≤ 93% at a resting state; arterial partial pressure of oxygen/oxygen concentration ≤ 300 mmHg) and critical (occurrence of respiratory failure requiring mechanical ventilation; presence of chock; other organ failure that requires monitoring and treatment in the ICU) according to the severity of clinical symptoms, imaging manifestations and hypoxia.

Venous blood sampling was performed immediately upon hospitalization, and the white blood cell count (WBC), neutrophil count (NEU), lymphocyte count (LYM), and procalcitonin (PCT) and C-reactive protein (CRP) levels were determined using standard laboratory methods. Consistent laboratory methods were adopted.

### Clinical Outcome Ascertainment

The primary endpoint was the conversion time from the first positive of SARS-CoV-2 RNA test to negative in respiratory tract samples. Negative conversion of SARS-CoV-2 RNA was defined as undetectable viral RNA for two consecutive respiratory tract or fecal samples (sampling interval more than 24 h). The secondary outcome was the time of hospitalization for COVID-19. Discharge standards were defined as follows: (1) body temperature remaining normal (axilla temperature ≤ 36.6°C or oral temperature ≤ 37.2°C or rectal temperature ≤ 37.8°C) for at least 3 days; (2) respiratory symptoms improved, SpO_2_ >93% without assisted oxygen inhalation; (3) lung imaging showing obvious improvement in lesions, CT improvement defined as that exudation or consolidation of the lesion are absorbed, the lesion area was gradually narrowed, and there may be residual linear fibrosis, which are independently judged by two radiologist until they reached the same conclusion; (4) negative conversion of SARS-CoV-2 RNA in respiratory tract and fecal samples; (5) disease course at least 14 days from onset; and (6) discharge approved by multi-disciplinary medical team.

### Statistical Analysis

Statistical analyses were performed using SPSS Statistics version 25.0. Basic characteristics of the study subjects are presented as the median (range) for continuous variables and as numbers with percentages for categorical variables. Independent *t*-tests or the Mann–Whitney U tests were performed for comparison of means of continuous variables depending on the normality of the distribution of groups. Paired-samples *t-*test was adopted to compare means of paired samples. For venous blood results, values beyond the defined lower limit of detection were set to the lower limit. The Pearson χ2 test was used to assess differences in categorical variables between groups. The log-rank test and Kaplan-Meier curve analyses were performed to compare the duration of SARS-CoV-2 shedding and hospitalization between groups. A Cox proportional hazard model was applied to estimate the HRs and corresponding 95% CIs. Subgroup analysis including sex, age, epidemiological history, clinical classification and antiviral treatment was carried out to adjust for potential imbalance in baseline characteristics. For all comparative analyses, *P* < 0.05 was considered to indicate statistical significance.

## Results

### Characteristics of the Study Population

This study included a total of 104 patients with COVID-19, with 54 (51.9%) in the infection group and 50 (48.1%) in the non-infection group. The characteristics of the study population are listed in Table 1. A significant difference in the sex was found between the groups (*P* = 0.002). There were 34 male patients (63.0%) in the infection group and 16 male patients (32%) in the non-infection group. Compared with patients without SARS-CoV-2 GI infection, a significantly higher likelihood of GI infection was found for overseas cases (8 (14.8%) vs. 1 (2.0%); *P* = 0.020). Among these overseas cases with GI infection, 5 patients were from the UK, 2 patients from Hong Kong, and 1 patient from Macau. In contrast, age, ethnicity, smoking, drinking, medical history, and clinical classification were not significantly different between the groups. We also observed no differences in the WBC, NEU, LYM, CRP and PCT levels between the two groups (Table 1).

**Table 1:** Characteristics of the study population.

	**All patients**	**Infection**	**Non-infection**	***P*-value**
	**(*n* = 104)**	**(*n* = 54)**	**(*n* = 50)**	
Sex				0.002
Male, %	50, 48.1	34, 63.0	16, 32.0	
Female, %	54, 51.9	20, 37.0	34, 68.0	
Age group				0.637
>65, %	12, 11.5	7, 13.0	5, 10.0	
≤ 65, %	92, 88.5	47, 87.0	45, 90.0	
Ethnicity				0.389
Asian, %	102, 98.2	52, 96.4	50, 100.0	
Caucasian, %	1, 0.9	1, 1.8	0, 0.0	
Black, %	1, 0.9	1, 1.8	0, 0.0	
Smoking, %	6, 5.8	3, 5.6	3, 6.0	0.923
Drinking, %	2, 1.9	1, 1.9	1, 2.0	0.956
Epidemiological history				0.020
Local cases, %	95, 91.3	46, 85.2	49, 98.0	
Overseas cases, %	9, 8.7	8, 14.8	1, 2.0	
Medical history				
Hypertension, %	18, 17.3	8, 14.8	10, 20.0	0.485
Diabetes, %	7, 6.7	2, 3.7	5, 10.0	0.200
Clinical classification				0.129
Mild, %	14, 13.4	10, 18.5	4, 8.0	
Moderate, %	66, 63.5	29, 53.7	37, 74.0	
Severe, %	19, 18.3	11, 20.4	8, 16.0	
Critical, %	5, 4.8	4, 7.4	1, 2.0	
Antiviral treatment, %	93, 89.4	48, 88.9	45, 90.0	0.854
WBC (10∧9/L)^*^	4.92	4.97	4.75	0.689
	(0.19–24.72)	(0.19–14.95)	(2.62–24.72)	
NEU (10∧9/L)^*^	2.82	2.94	2.81	0.930
	(0.46–10.63)	(0.46–10.63)	(0.95–7.43)	
LYM (10∧9/L)^*^	1.52	1.55	1.51	0.738
	(0.21–9.02)	(0.21–9.02)	(0.58–4.19)	
CRP (mg/L)^*^	3.67	2.39	5.35	0.224
	(0.01–115.14)	(0.01–74.11)	(0.03–115.14)	
PCT (ng/mL)^*^	0.10	0.10	0.10	0.056
	(0.10–5.75)	(0.10–5.75)	(0.10–0.30)	

*WBC, white blood cell count; NEU, neutrophil count; LYM, lymphocyte count; CRP, C-reactive protein; PCT, procalcitonin.*

* *median (range)*.

### Clinical Outcome of Patients With SARS-CoV-2 GI Infection

**Figure 1:**
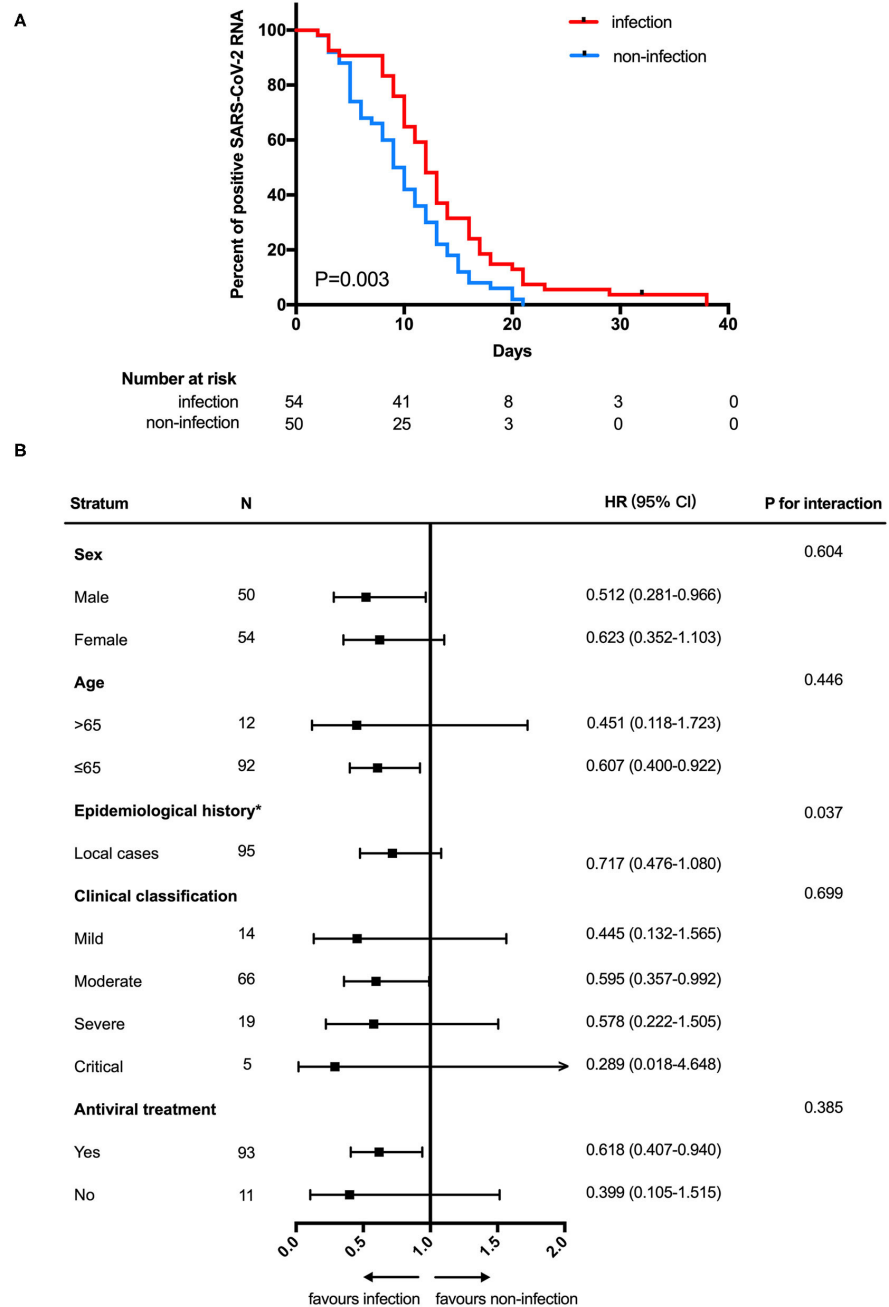
Negative conversion time **(A)** Kaplan-Meier curve for the duration of SARS-CoV-2 RNA in respiratory tract samples between SARS-CoV-2 gastrointestinal infection and non-infection groups. **(B)** Subgroup analysis. NCT, negative conversion time; HR, hazard ratio. *The number of patients in the overseas case subgroup was too few for comparison.

**Figure 2:**
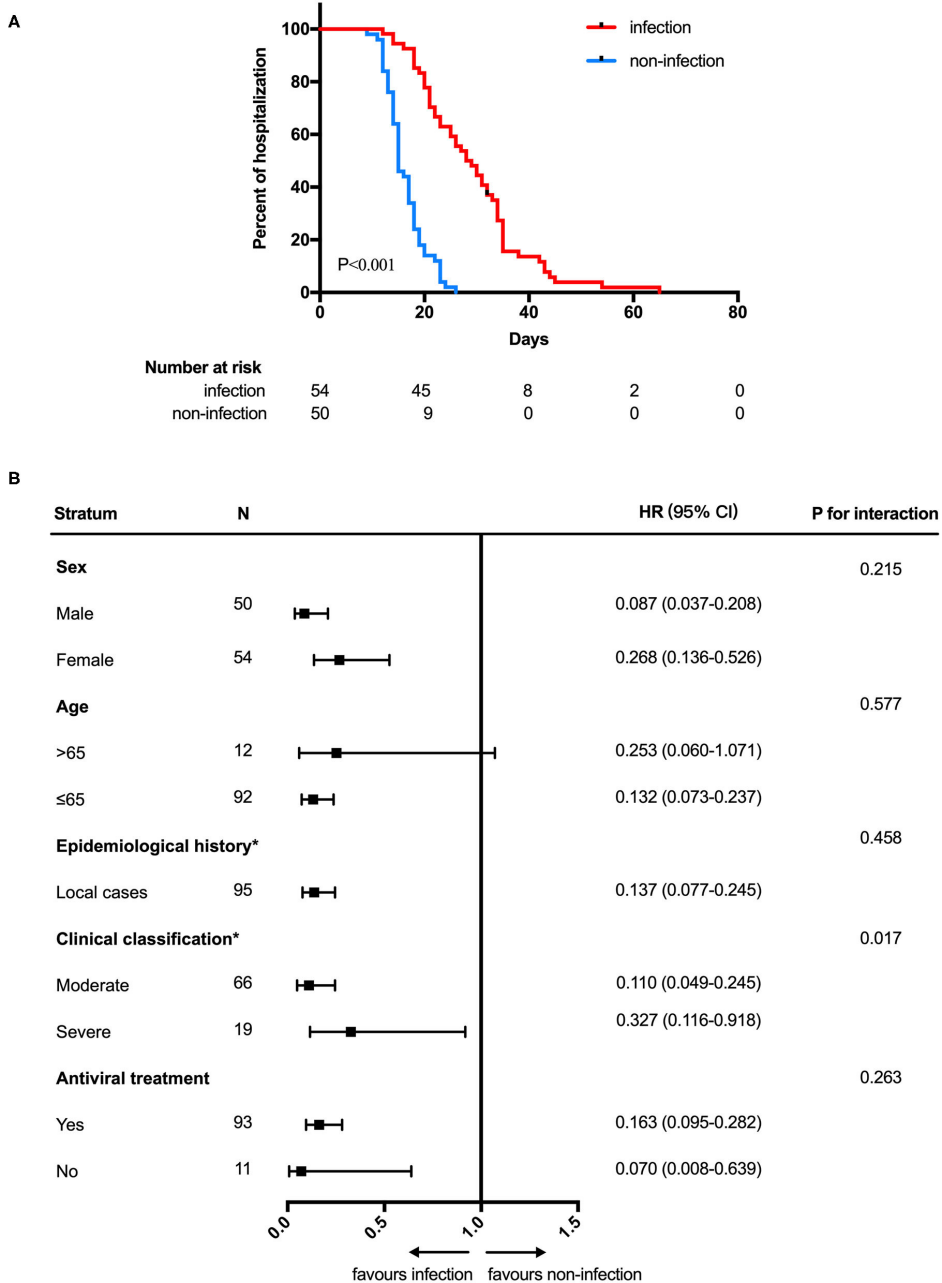
Hospitalization time **(A)** Kaplan-Meier curve for hospitalization time between SARS-CoV-2 gastrointestinal infection and non-infection groups. **(B)** Subgroup analysis. HOD, hospitalization days; HR, hazard ratio. *The number of patients in the overseas case subgroup as well as the mild and critical subgroups were too few for comparison.

In analyses of the full study population, patients with SARS-CoV-2 GI infection exhibited a longer duration of SARS-CoV-2 RNA positivity in respiratory tract samples than patients without SARS-CoV-2 GI infection (median 12.0 days [95% CI: 10.0–13.2] vs. 9.0 days [95% CI: 7.5–10.5]; HR 0.575 [95% CI: 0.386–0.857]; *P* = 0.003) (Figure 1A). We also found that patients with SARS-CoV-2 GI infection had a longer time of hospitalization than the patients without SARS-CoV-2 GI infection (median 28.0 days [95% CI: 23.2–32.8] vs. 15.0 days [95% CI: 13.6–16.4]; HR 0.149 [95% CI: 0.087–0.252]; *P* < 0.001) (Figure 2A). To adjust for potential imbalance in the baseline characteristics of the two groups, which is inevitable in a retrospective study, subgroup analyses of sex, age, epidemiological history, clinical classification and antiviral treatment were performed. Combined SARS-CoV-2 GI and respiratory infection had an adverse effect on the time to negative conversion of viral RNA in respiratory tract samples as well as hospitalization time in all strata (Figures 1B, 2B). Nonetheless, the effect was not statistically significant in some subgroups because of the small sample size.

### Evaluation of the Clinical Characteristics of Patients With Persistently Positive SARS-CoV-2 RNA in Feces

Among the GI infection group, we specially evaluated the viral load of 16 patients who remained persistently positive fecal SARS-CoV-2 RNA over nearly 2 weeks or more. Among the 16 patients, SARS-CoV-2 RNA in feces was detected 1–21 days (median: 6 days; mean: 8.44 days) after detection of viral RNA in nasopharyngeal swabs. The mean duration of positive fecal SARS-CoV-2 RNA was significantly longer than that of positive nasopharyngeal SARS-CoV-2 RNA (25.81 days vs. 9.88 days; difference in mean 15.94 [95% CI: 11.93–19.95]; *P* < 0.001) (Table 2).

**Table 2:** Clinical characteristics of patients with persistently positive SARS-CoV-2 RNA in feces.

**Patient**	**Sex**	**Age (years)**	**Interval between initial detection of positive N and F (days)**	**Duration of positive N (days)**	**Duration of positive F (days)**	**SARS-CoV-2 nucleocapsid protein in gastric epithelial cells**
patient 1	M	29	6	7	26	/
patient 2	M	35	16	13	12	/
patient 3	M	1.4	9	6	29	/
patient 4	F	29	6	10	29	/
patient 5	F	34	6	15	28	Negative
patient 6	M	32	8	10	26	/
patient 7	M	44	5	15	27	/
patient 8	M	42	6	3	27	/
patient 9	F	53	4	10	25	/
patient 10	M	45	1	1	29	/
patient 11	M	65	14	18	28	Positive
patient 12	F	0.9	5	8	28	/
patient 13	M	38	21	7	28	/
patient 14	F	63	21	14	17	/
patient 15	M	64	6	13	27	Positive
patient 16	M	64	1	8	27	/
Median	NA	40	6	10	27	/
Mean	NA	39.96	8.44	9.88	25.81	/
Max	NA	65	21	18	29	/
Min	NA	0.9	1	1	12	/

*N, nasopharyngeal swab; F, feces/no data; NA, not applicable*.

**Figure 3:**
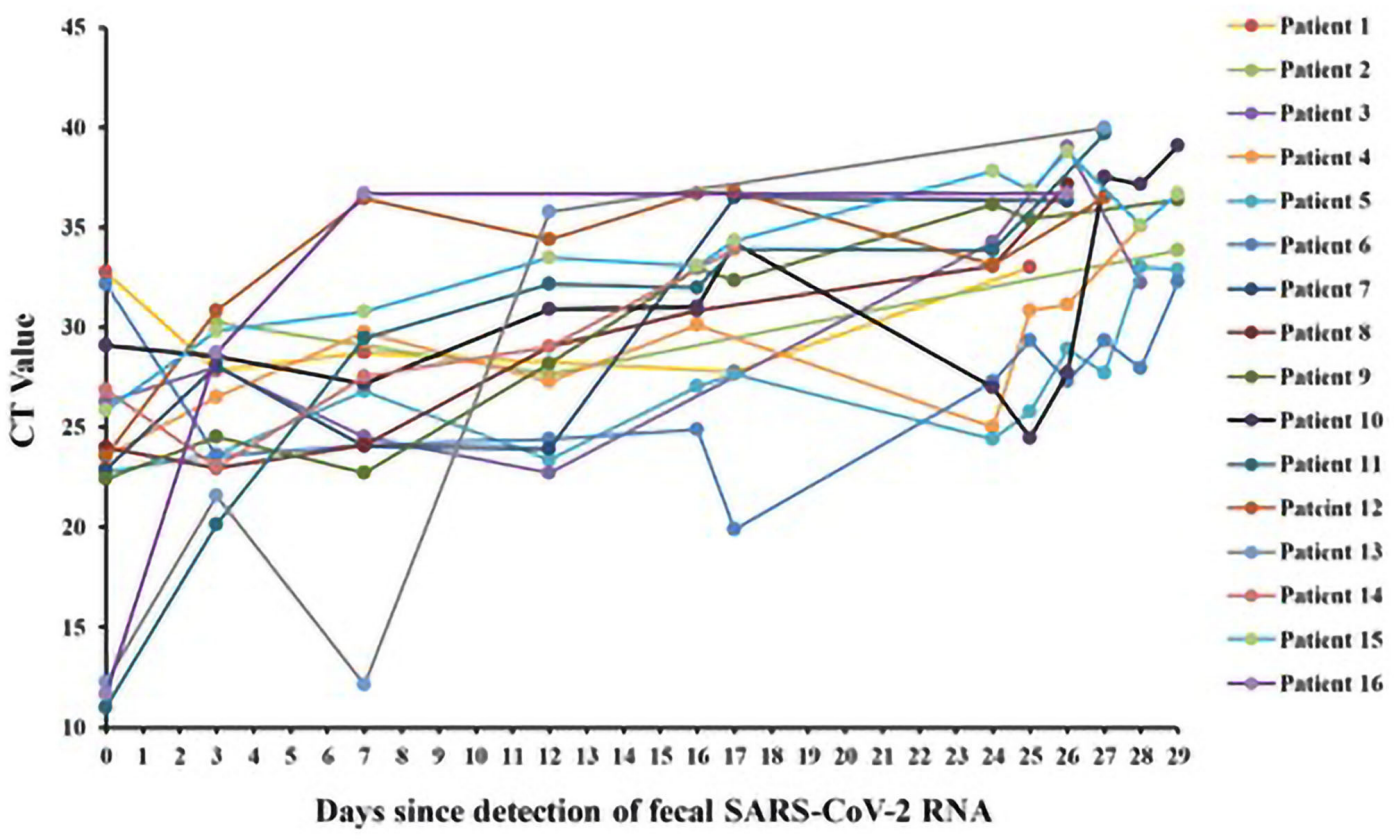
CT values of fecal SARS-CoV-2 RNA in patients with persistently positive SARS-CoV-2 RNA in feces (*n* = 16).

We monitored CT value of fecal sample from 16 patients during their hospitalization. As shown in Figure 3, CT values of the fecal sample ranged from 11 to 40. The mean of the initial and the final CT values is 23.59 and 36.22, respectively (difference in mean −12.62 [95% CI: −18.67 to −6.579]; *P* < 0.001). Except for Patient 11, 13, and 16, the CT values of the other 13 patients increased slowly over time during their hospitalization. The CT values of Patient 1 and 6 increased <1 over 25 and 29 days.

Among 3 patients (Patient 5, Patient 11, and Patient 15) undergoing upper GI endoscopy, SARS-CoV-2 nucleocapsid was detected in the cytoplasm of gastric glandular epithelial cells in 2 patients (Patient 11 and 15), who remained positive for fecal SARS-CoV-2 RNA after negative conversion of viral RNA from pharyngeal swabs (Table 2).

## Discussion

SARS-CoV-2 binds to angiotensin-converting enzyme 2 (ACE2) to enter host cells via its surface spike glycoprotein. ACE2 is highly expressed in GI epithelial cells and mediates SARS-CoV-2 entry, leading to GI infection and corresponding GI symptoms (13–15).

Although GI infection has received attention, there is still a lack of in-depth and comprehensive understanding of the clinical characteristics of SARS-CoV-2 GI infection. In the present study, we explored the impact of GI infection with SARS-CoV-2 on the clinical outcomes of COVID-19 and found that it prolongs the duration of SARS-CoV-2 shedding and hospitalization in patients with COVID-19.

51.9 percentage of patients with COVID-19 (54/104) had GI infection of SARS-CoV-2 in our study, and these patients tended to be male. Previous studies on COVID-19 also found that males might be more susceptible to SARS-CoV-2, with more serious medical conditions and higher mortality (16–19). This might be because of the robust T cell response in female patients (20) and protection against the virus by female hormones (21, 22). This finding also provides a possible explanation for the observed sex biases in the SARS-CoV-2 GI infection. Additionally, a significant difference in epidemiological history between the infection and non-infection groups was observed in our study. A previous study subdivided the global SARS-CoV-2 population into six well-defined subtypes by focusing on widely shared polymorphisms in non-structural cistrons and structural and accessory genes (23). Indeed, cases from different regions may be infected with different subtypes of the virus, which suggests that patients with COVID-19 in some regions may have higher morbidity of GI infection. However, this phenomenon needs to be confirmed, and its mechanism should be further explored.

We explored the relationship between clinical outcomes of COVID-19 and GI SARS-CoV-2 infection which defined as simultaneously positivity for SARS-CoV-2 RNA in a fecal sample. Several studies have evaluated the relationship between GI manifestations and clinical outcomes of COVID-19 and found that the presence of GI manifestations does not appear to affect mortality of COVID-19 (9–11). GI symptoms are common in viral infections and no specificity, which is not equal to GI infection. In this study, we found that SARS-CoV-2 GI infection led to a longer duration of SARS-CoV-2 RNA presence in respiratory tract samples and longer hospitalization time, which will result in more medical and financial investments. Because of the imbalance in the baseline characteristics of the two groups in this study, subgroup analyses of sex, age, epidemiological history, clinical classification and antiviral treatment were performed and we found that the adverse effect of SARS-CoV-2 GI infection on the duration of SARS-CoV-2 RNA in respiratory tract samples and hospitalization time to be consistent across subgroups. Evaluation of viral load for persistently SARS-CoV-2 GI infection suggested that the initial viral load of GI infection was high and decreased slowly. Nevertheless, detection of SARS-CoV-2 nucleocapsids in gastric glandular epithelial cells in 2 patients with positive feces and negative nasopharyngeal swabs indicated that SARS-CoV-2 GI infection persisted. The GI system is an appropriate intrusion portal and is a potential virus pool, which may prolong the clinical course and influence the clinical outcomes of COVID-19. Regardless, due to the small sample of this study, larger-sample, multicenter and prospective studies should be undertaken to determine the effect.

The present study has several limitations. First, this was a small-sample, single-center, retrospective observational study, and the included population lacked satisfactory representation, even though it covered all age and ethnicity groups. Second, the discharge standards were based on handbook of COVID-19 prevention and treatment (in Chinese), which contained some subjective items and may interfere with the evaluation of the importance of SARS-CoV-2 GI infection in clinical outcomes of COVID-19. In the present study, discharge approved by fixed medical team, which minimized the influence of personal subjective factors on the results as much as possible. Moreover, it is not completely accurate to determine GI infection by detecting SARS-CoV-2 RNA in a fecal sample using rRT-PCR, and some patients with SARS-CoV-2 GI infection may have a negative rRT-PCR and be excluded from the study; thus, further study with more accurate testing methods and diagnostic criteria should be undertaken.

In conclusion, this study explored the impact of SARS-CoV-2 GI infection on clinical outcomes of COVID-19. About half of patients with COVID-19 have GI infection of SARS-CoV-2, and male patients and overseas cases may be more susceptible to GI infection. GI infection with SARS-CoV-2 had a high viral load and prolonged the duration of SARS-CoV-2 shedding as well as hospitalization in patients with COVID-19, which will cause more medical and financial investments. Therefore, it is necessary to pay more attention to SARS-CoV-2 GI infection and fecal SARS-CoV-2 RNA test should be completed in time.

## Data Availability Statement

The original contributions presented in the study are included in the article/supplementary material, further inquiries can be directed to the corresponding author/s.

## Author Contributions

All authors listed have made a substantial, direct and intellectual contribution to the work, and approved it for publication.

## Conflict of Interest

The authors declare that the research was conducted in the absence of any commercial or financial relationships that could be construed as a potential conflict of interest.
